# Supplementary material for: Sleep health of Australian community tennis players during the COVID-19 lockdown
Source: PeerJ. 2022 Apr 12;10:e13045. doi: 10.7717/peerj.13045 (PMC9012171; doi:10.7717/peerj.13045)
Supplement: Supplemental Information 2 [file peerj-10-13045-s002.pdf]

Q1 – In general, how would you rate your sleep quality?

Options to select from: very good, good, fair, poor

Q2 – Thinking about just the past 7 days, what time did you most often go to bed on work days? If you did not work last week, please answer about weekdays. [please report 24 hour time; e.g. 2200]

Q3 – What about on non-work days or weekends – what time did you most often go to bed on those days? [please report 24 hour time; e.g. 2200]

Q4 – What time did you most often wake up for the day on work days or weekdays? [please report 24 hour time; e.g. 0600]

Q5 – What about on non-work days or weekends – what time did you most often wake up for the day on those days? [please report 24 hour time; e.g. 0600]

Q6 – During the past 7 days, how many days did you wake up feeling well-rested, if any?

Options to select from: 1, 2, 3, 4, 5, 6, 7, Don't know

Q7 – How many nights did you have trouble falling asleep?

Options to select from: 1, 2, 3, 4, 5, 6, 7, Don't know

Q8 – And how many nights did you have trouble staying asleep?

Options to select from: 1, 2, 3, 4, 5, 6, 7, Don't know

Q9 – Still thinking about the past 7 days, how many days did poor or insufficient sleep significantly impact your daily activities, like your work performance, socializing, exercising, or other typical activities?

Options to select from: 1, 2, 3, 4, 5, 6, 7, Don't know

Q10 – How many days did you fall asleep without intending to, such as dozing in front of the TV or in any other situation?

Options to select from: 1, 2, 3, 4, 5, 6, 7, Don't know

Q11 – How many nights did you take over-the-counter or prescription medication to help you sleep?

Options to select from: 1, 2, 3, 4, 5, 6, 7, Don't know

Q12 – Have you ever been told by a doctor that you have a sleep disorder, such as insomnia or sleep apnea, or not?

Options to select from: Yes, No, Don't know

Q13 – Have you ever discussed any sleep problems you were having with a doctor or medical professional?

Options to select from: Yes, No, Don't know

Q14 – How many hours of sleep do you need per day to be well-rested and feel your best?

Options to select from: 1, 2, 3, 4, 5, 6, 7, 8, 9, 10, 11 ,12 ,13, 14, 15, 16, 17, 18, 19, 20, 21, 22, 23, 24, Don't know

Figure S1: Sleep Health Index questions.

Q1 – How satisfied are you with your sleep overall?

Options to select from: Very satisfied, Satisfied, Dissatisfied, Very dissatisfied

Q2 – Generally speaking, when you wake up in the morning, how refreshed do you feel?

Options to select from: Very refreshed, Refreshed, Hardly refreshed, Not refreshed at all

Q3 – Based on how well you sleep, how energized do you generally feel as you go about your day?

Options to select from: Very energised, Energised, Hardly energised, Not energised at all

Q4 – How much trouble do you generally have falling asleep, if any?

Options to select from: No trouble at all, A little trouble, A fair amount of trouble, A lot of trouble

Q5 – How often do you wake up during the night for any reason?

Options to select from: Almost always, Often, Sometimes, Rarely, Never

Q6 – If you wake up after falling asleep, is it generally easy or difficult to fall back asleep?

Options to select from: Very difficult, Difficult, A little difficult, Not difficult

Q7 – In general, how satisfied are you with the amount of sleep you get on work-days or weekdays?

Options to select from: Very satisfied, Satisfied, Dissatisfied, Very Dissatisfied

Q8 – In general, how satisfied are you with the amount of sleep you get on weekends or non-work days?

Options to select from: Very satisfied, Satisfied, Dissatisfied, Very dissatisfied

Q9 – How easy or difficult is it for you to achieve a relaxed mental state before going to bed – is this… very difficult, difficult, a little difficult, not difficult at all?

Options to select from: Very difficult, Difficult, A little difficult, Not difficult at all

Figure S2: Sleep Satisfaction Tool questions.

Tennis play:

Q1 – On average, since the COVID-19 situation how many hours a week are you training tennis?

Options to select from: 1, 2, 3, 4, 5, 6, 7, 8, 9, 10, 11, 12, 13, 14, 15, 16, 17, 18, 19, 20, More than 20

Physical activity:

Q1 – On average, since the COVID-19 situation how many hours a week are you training strength?

Options to select from: 1, 2, 3, 4, 5, 6, 7, 8, 9, 10, 11, 12, 13, 14, 15, 16, 17, 18, 19, 20, More than 20

Q2 – On average, since the COVID-19 situation how many hours a week are you training endurance?

Options to select from: 1, 2, 3, 4, 5, 6, 7, 8, 9, 10, 11, 12, 13, 14, 15, 16, 17, 18, 19, 20, More than 20

Q3 – On average, since the COVID-19 situation how many hours a week are you training speed & agility?

Options to select from: 1, 2, 3, 4, 5, 6, 7, 8, 9, 10, 11, 12, 13, 14, 15, 16, 17, 18, 19, 20, More than 20

Training location:

Q1 – Where are you performing your physical training?

Options to select from: I'm not performing any physical training during the COVID-19 situation, Home, Local park, Sporting club, Others (Please specify)

Alcohol consumption:

Q1 – Since the COVID-19 situation how often do you drink alcohol?

Options to select from: Few times a week, Once a week, Once every two weeks, Once a month, Less than once a month, I don't drink alcohol at all

Figure S3: Questions about lifestyle factors.
